# Evaluation of skeletal muscle elasticity using color Doppler shear wave imaging

**DOI:** 10.1007/s40477-023-00795-3

**Published:** 2023-06-21

**Authors:** Yuji Kanaya, Kei Konno, Yoshiki Yamakoshi, Nobuyuki Taniguchi, Hideaki Watanabe, Katsushi Takeshita

**Affiliations:** 1 Department of Orthopaedic Surgery, Jichi Medical University, 3311-1 Yakushiji, Shimotsuke, Tochigi 329-0498 Japan; 2 Department of Clinical Laboratory Medicine, Jichi Medical University, 3311-1 Yakushiji, Shimotsuke, Tochigi 329-0498 Japan; 3 Gunma University Graduate School of Science and Technology, Gunma University, 1-5-1 tenjin-cho, kiryu, Gunnma 376-8515 Japan

**Keywords:** Color Doppler shear wave imaging, Shear wave elastography, Skeletal muscle, Tissue elasticity

## Abstract

**Purpose:**

This study aimed to (1) assess the precision and reproducibility of color Doppler shear wave imaging (CD SWI) by comparing it with shear wave elastography (SWE) via elasticity phantom measurements, and (2) investigate the potential clinical applications of CD SWI in the upper limb muscles by assessing the reproducibility of skeletal muscle elasticity evaluations.

**Methods:**

Four elastography phantoms of different stiffness (6.0–7.5 wt%) were used to assess the precision and reproducibility of CD SWI (compared with SWE) at depths. Typical upper limb muscles of 24 men were also assessed for this comparison.

**Results:**

At superficial depths (0–2 cm), the phantom measurements obtained using CD SWI and SWE were similar at all levels of stiffness. Furthermore, both methods were highly reliable, with almost perfect intra- and inter-operator reliabilities. At greater depths (2–4 cm), measurements obtained using both methods were similar at all stiffness levels. Although standard deviations (SDs) of the phantom measurements obtained using both methods at lower stiffness were similar, those at higher stiffness were different. The SD of the CD SWI measurements was < 50% of that of the SWE measurements. However, both methods were highly reliable in the phantom test, with almost perfect intra- and inter-operator reliabilities. The intra- and inter-operator reliabilities of the shear wave velocity measurements for typical muscles of the upper limbs were also substantial in clinical settings.

**Conclusion:**

CD SWI is a valid method for measuring elasticity, with precision and reliability as high as those of SWE.

**Graphical abstract:**

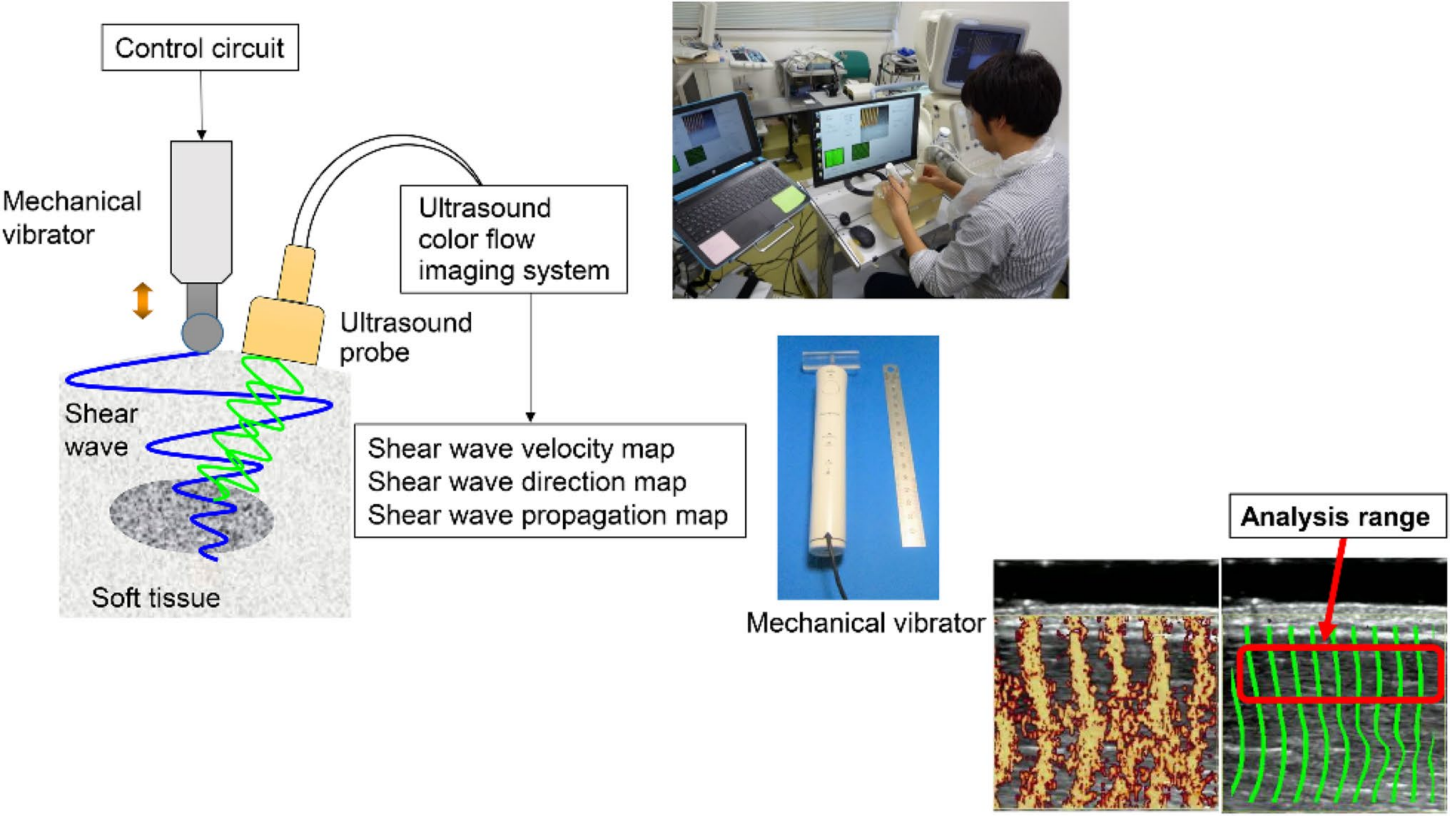

## Introduction

Various reports have been recently publishedon the use of elastographyto evaluate tissue elasticity. Elastography aids in the diagnosis of conditions related to the liver and mammary glands by quantitatively or qualitatively displaying physical findings or tactile phenomena observable by most doctors as unusual stiffness in fibrosis or malignant lesions [1, 2].

Many reports have been published in the field of orthopedics, including those evaluating the efficacy of physical therapy by measuring changes in muscle elasticity before and after rehabilitation [3] and exercise [4].

Shear wave elastography (SWE) measures the velocity of shear waves in the body, taking advantage of the fact that shear wave propagation velocity (SWV) is determined by tissue elasticity and is more quantitative than strain elastography (SE). Improved accuracy has been reported [5].

In SWE, which is currently the most commonly used form of elastography, acoustic radiation is generated using ultrasound [6]. However, concerns regarding ultrasound-induced tissue heating [7, 8] and damage [9] have been raised, and the cost of equipment remains high.

**Fig. 1:**
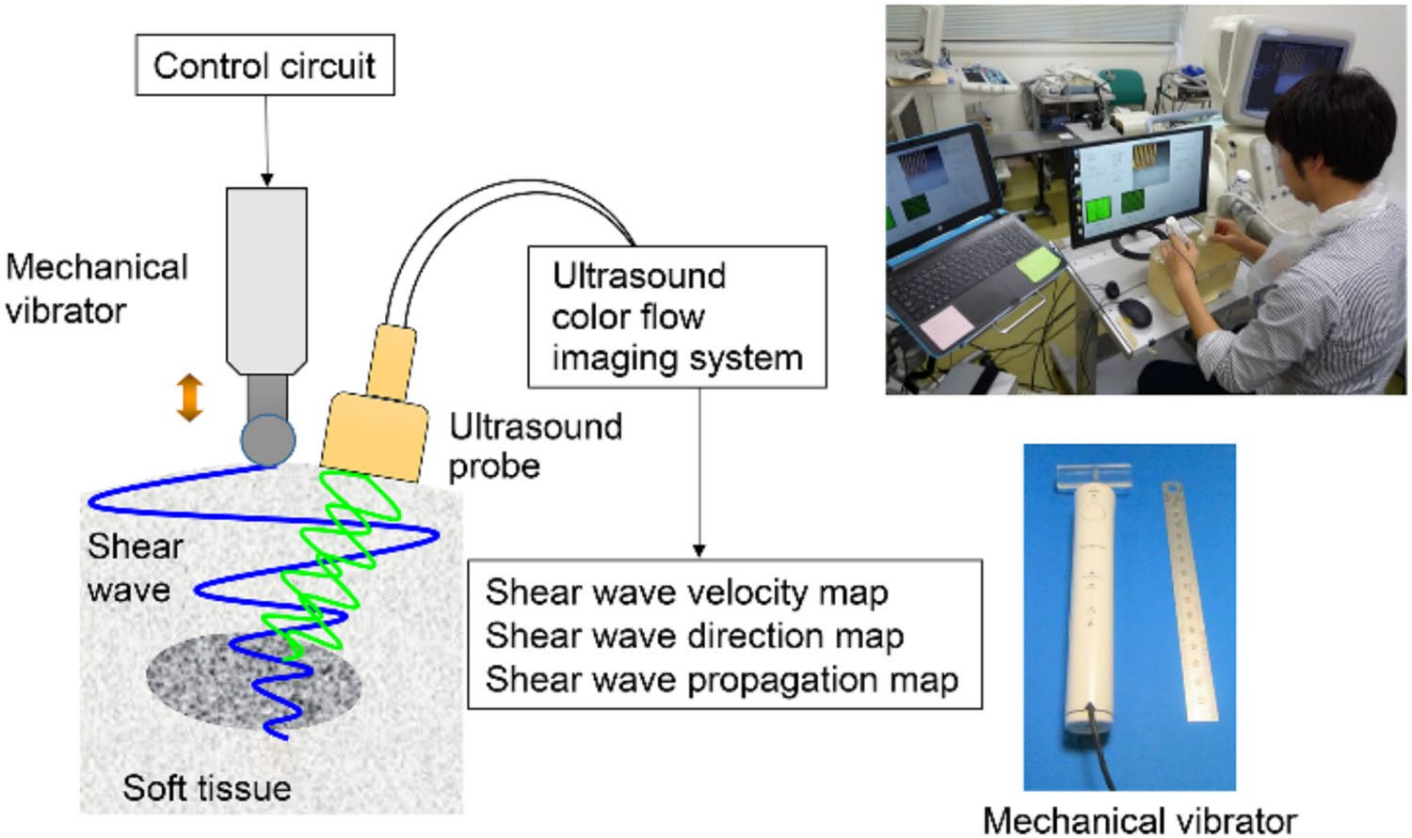
Experimental setup and picture of an example measurement

Amid such concerns, Yamakoshi et al. developed color Doppler shear wave imaging (CD SWI), a novel method for displaying shear waves, and reported on its usefulness [10–12]. In CD SWI, a continuous shear wave is produced by attaching a small ultrasonic actuator to the tissue surface and applying vibrations (Fig. 1).

In this context, the aims of the present study were to: (1) compare CD SWI and SWE to evaluate the precision of CD SWI and (2) perform a fundamental investigation of the clinical applications of CD SWI for locomotory organs through reproducible evaluation of skeletal muscle elasticity.

## Materials and methods

### Evaluation of the precision of CD SWI compared with that of SWE

#### Participants

**Fig. 2:**
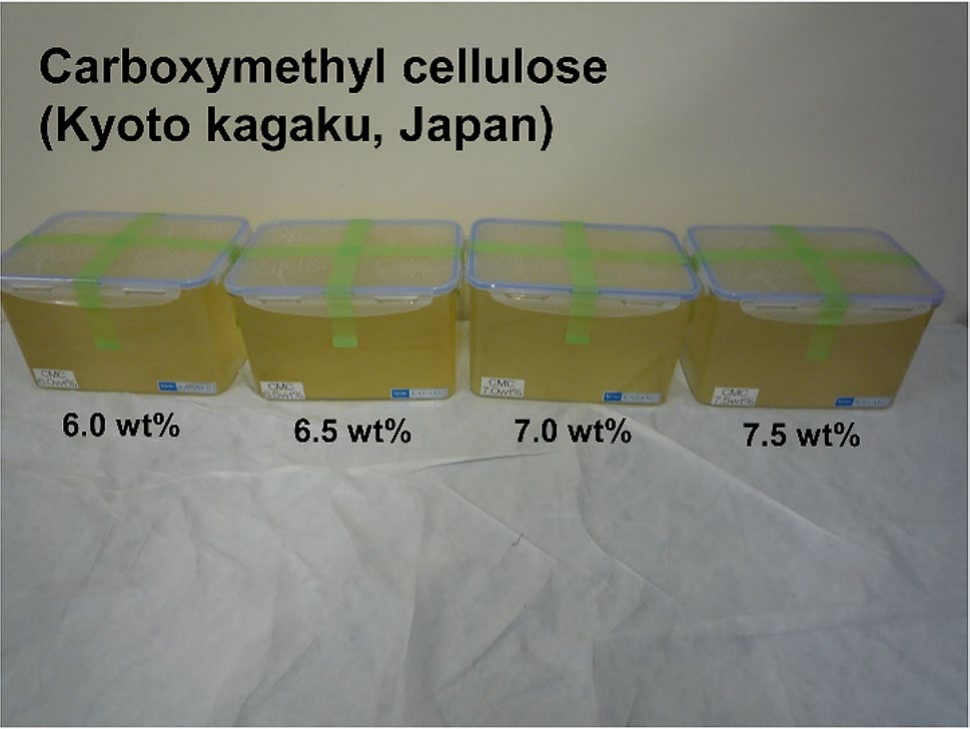
Carboxymethylcellulose samples of four different hardnesses used as phantom experiments

Four different internally homogeneous ultrasound elastography phantoms (Kyoto Kagaku Co., Ltd., Kyoto, Japan) containing 6.0, 6.5, 7.0, and 7.5 wt% carboxymethylcellulose were used as the research objects to compare the precisions of CD SWI and SWE (Fig. 2).

#### SWE measurement

SWV was measured using Aplio i800 (Canon, Tokyo, Japan).

#### SWV measurement using CD SWI

The operator held a probe at the center of the phantom being used for measurement with the right hand and applied vibration using an ultrasonic actuator held 1 cm away in the left hand to produce shear waves in the phantom, and the SWV was measured (Fig. 1).

#### SWV measurement

SWV in the phantoms was measured five times each in the superficial (0–2 cm) and deep (2–4 cm) layers, and the median value was selected as the measured value. Measurements were performed by two operators (I and II), using both CD SWI and SWE units. The same protocols were repeated under the same conditions on three separate days to obtain three sets of measurements. Before the measurements, the shear wave propagation velocity was calibrated with the SWE unit and the 6.0 wt% phantom.

### Evaluation of reproducibility in measuring skeletal muscle tissue elasticity

#### Participants

The participants were 24 men aged 20 years or older who consulted the orthopedic outpatient department of XXX University Hospital and presented with no symptoms or previous problems in their upper limbs. The mean age was 24 (21–26) years; mean height was 163.5 ± 5.72 cm; mean weight was 60.3 ± 6.1 kg; and mean body mass index was 22.48 ± 1.12 kg/m^2^. This study was approved by the XXX Clinical Research Ethics Committee (Approval Number XXX). All research participants provided written consent after receiving sufficient explanation.

#### Methods

The method described in the section *SWV measurement using CD SWI* was used for the measurement of the superficial (trapezius and biceps brachii) and deep (supraspinatus and brachialis) muscles of the non-dominant hand.

The identification and measurements of the trapezius (superficial) and supraspinatus (deep) muscles were performed as follows: Each participant was seated in a chair with the back of the hands resting on the mid-thighs. The acromia and spinous process of the seventh cervical vertebra were marked, and the trapezius was identified at a 1/3rd distance on a straight line connecting these two points; at the same location, the supraspinatus was also identified. For the measurements, the ultrasound probe was placed longitudinally to the muscle, and the actuator was placed 1 cm toward the trunk to vibrate the muscle and excite shear waves.

The biceps brachii (superficial) and brachialis (deep) muscles were identified and measured as follows. Each participant was instructed to lie in the supine position on a bed with the backs of their hands and wrists resting on the bed. The coracoid process was marked, the distal biceps tendon was marked at the radial tuberosity, and the point halfway along a line connecting these two points was identified. An ultrasound probe was then placed on the upper arm. The wrist was pronated and supinated while observing the longitudinal B-mode image, in which the moving superficial muscle was identified as the biceps brachii and the stationary deeper muscle was identified as the brachialis. The ultrasound probe was placed longitudinally to the muscles, with an oscillator placed 1 cm away to excite the shear wave.

#### SWV measurements in muscles

**Fig. 3:**
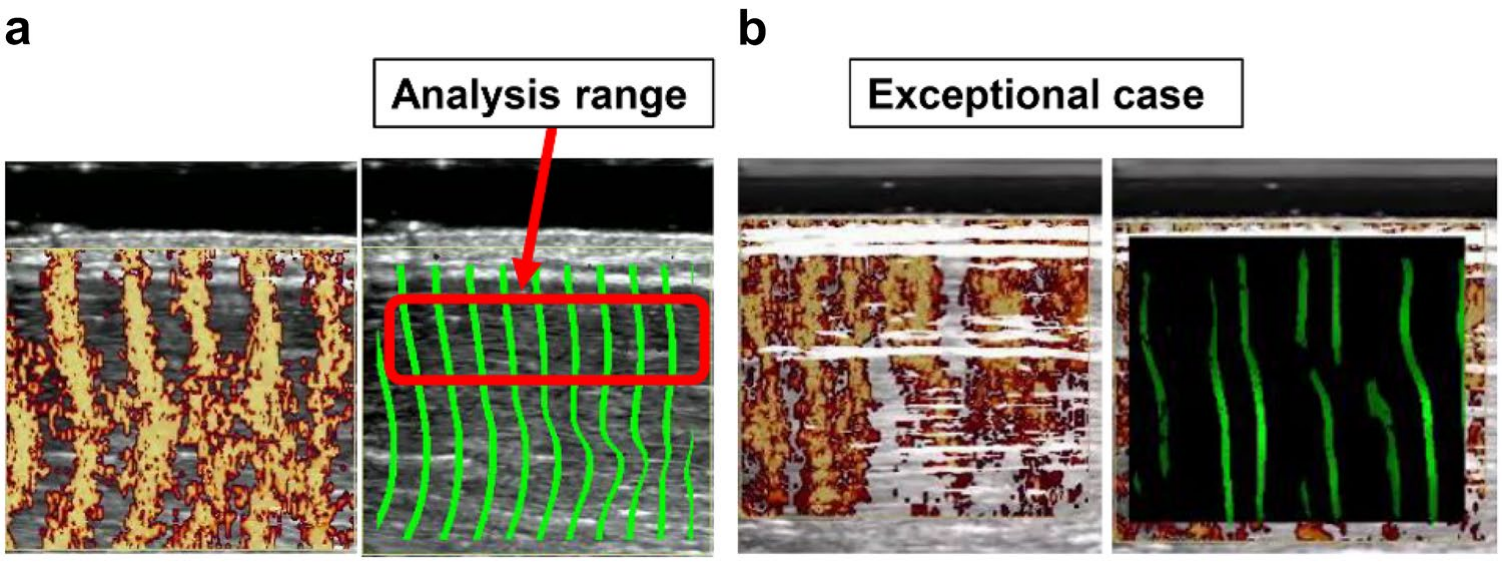
An example of a shear wave propagation image by CD SWI in phantom experiments. **a** Clear shear wave propagation worth analyzing. **b** The shear wave is not clearly mapped compared to (**a**). This indicates that the shear waves are not propagating correctly in the material, so we excluded analyzing with these shear wave propagation images

Before measurement, the placement of the probe and oscillator was carefully adjusted, and both were held (without exerting pressure on the skin) to ensure that shear waves passing through the muscle were clearly displayed. The velocity of the shear wave passing through the various muscles was measured five times while maintaining the same conditions, and the median value was selected as the measured value. Measurements were performed by two operators (I and II) and were repeated on 3 separate days under the same conditions to obtain three sets of measurements. Measurements were selected for use when the images of the shear wave were sufficiently clear to be used for illustration and excluded when the wavefront was distorted (Fig. 3).

#### Assessment of the reproducibility and precision of measurements

Intraclass correlation coefficients (ICCs) were used to assess the reliability of measurements. Three sets of measurements obtained by the two operators were used to calculate ICCs (1.1) for assessing intra-operator reliability. Inter-operator reliability was also assessed using ICC (2.1). Simultaneously, the coefficient of variation (CV) was calculated to assess precision.

### Statistical analysis

Statistical analyses were performed using IBM SPSS Statistics, version 22 (IBM Japan, Tokyo, Japan). ICCs were interpreted according to the Landis and Koch classification [13], with 0.0–0.2, 0.21–0.40, 0.41–0.60, 0.61–0.80, and 0.81–1.0 indicating slight, fair, moderate, substantial, and almost perfect agreement, respectively.

## Results

### Precision of CD SWI compared with that of SWE

**Fig. 4:**
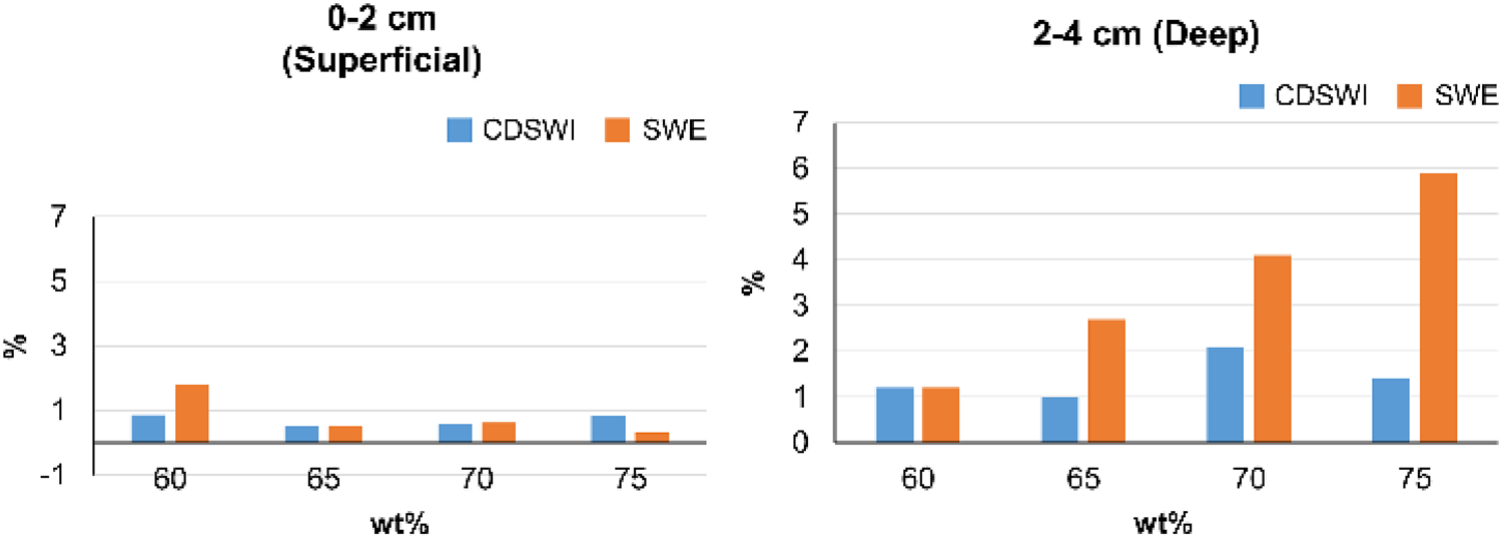
Coefficient of variation (CV) for each hardness phantom in the superficial (0–2 cm) and deep (2–4 cm) layers. In the superficial layers, the CV by CD SWI and SWE values were < 2%

The values obtained for the superficial and deep layers in the phantoms using CD SWI and SWE, along with their precision and reliability, are shown in Tables 1 and 2 and Fig. 4.

**Table 1:** Shear wave velocity (SWV) of the superficial and deep layers in phantoms calculated using color Doppler shear wave imaging (CD SWI) and shear wave elastography (SWE)

Phantom	SWV (m/s)	
	Superficial layer (0–2 cm)	Deep layer (2–4 cm)
6.0 wt%	C: 2.71 ± 0.02	C: 2.64 ± 0.02
	S: 2.71 ± 0.01	S: 2.91 ± 0.03
6.5 wt%	C: 2.98 ± 0.01	C: 2.91 ± 0.07
	S: 2.90 ± 0.01	S: 3.07 ± 0.08
7.0 wt%	C: 3.03 ± 0.01	C: 3.09 ± 0.08
	S: 3.05 ± 0.01	S: 3.17 ± 0.13
7.5 wt%	C: 3.26 ± 0.03	C: 3.19 ± 0.05
	S: 3.20 ± 0.01	S: 3.20 ± 0.19

6.0 wt% phantom was used for calibration

*C* CD SWI, *S* SWE

**Table 2:** Intra- and inter-operator reliability calculated using color Doppler shear wave imaging (CD SWI) and shear wave elastography (SWE)

Layer	C (CD SWI) ICC (1.1)	S (SWE) ICC (1.1)	ICC (2.1)
Superficial layer (0–2 cm)	i: 0.976	i: 0.986	C: 0.996
	ii: 0.990	ii: 0.994	S: 0.965
Deep layer (2–4 cm)	i: 0.889	i: 0.897	C: 0.996
	ii: 0.936	ii: 0.960	S: 0.990

i: observer 1, ii: observer 2

*C* CD SWI, *S* SWE

In the deep layers, there was a greater difference with stiffer phantoms in the SWE.

#### Superficial layer (0–2 cm)

The values obtained with the CD SWI were equivalent to those obtained using SWE at each level of stiffness. Both CD SWI and SWE showed increased SWV, accompanied by increased phantom stiffness (Table 1); the CV of each was < 2%. The intra- and inter-operator reliabilities of the CD SWI and SWE measurements were almost perfect for all measurements (Table 2). The results showed that CD SWI measures SWV at superficial depths with a precision and reliability similar to SWE.

#### Deep layer (2–4 cm)

Like in the superficial layers, in the deep layers, the values obtained with CD SWI were equivalent to those obtained with SWE at all levels of stiffness. Both CD SWI and SWE showed increased SWV, accompanied by increased phantom stiffness (Table 1). Although the CV was the same for CD SWI and SWE in the softest phantom, a greater difference was observed for stiffer phantoms. In the 7.0 wt% phantom, the CD SWI and SWE measurements showed CVs of 2.1% and 4.1%, respectively; in the 7.5 wt% phantom, the CVs were 1.4% and 5.9%, respectively, and the CV of the CD SWI measurements was less than half of that of the SWE measurements (Fig. 4). Both methods displayed almost perfect intra- and inter-operator reliabilities (Table 2).

Overall, the results of the phantom analysis showed that CD SWI can be used to measure SWV with precision and reliability equal to those of CD SWI at superficial depths. Although a drop in precision was observed with SWE when stiff phantoms were used, the precision of the CD SWI remained high.

### Reproducibility in measuring skeletal muscle elasticity

**Fig. 5:**
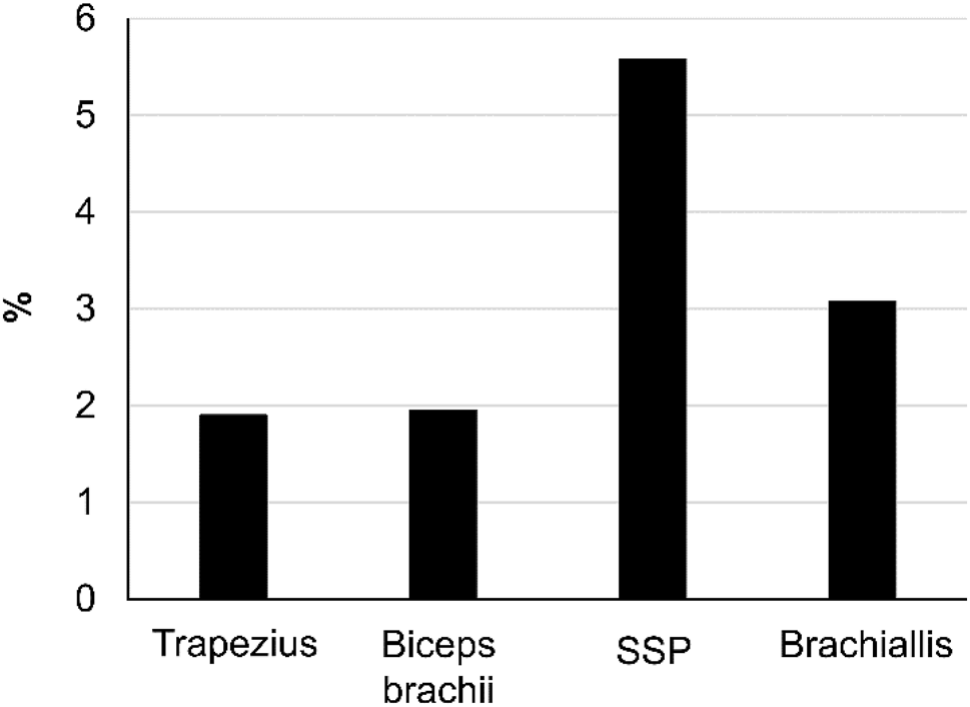
CV in the Trapezius, Biceps brachii, Supraspinatus (SSP), and Brachiallis was 2–5% on CD SWI

SWV with CD SWI was 3.6–3.8 m/s in all cases, which matches previous reports describing SWV in the same muscles [14–16]. The CV was 2–5%, with a tendency for the CV to be greater in measurements of the supraspinatus and brachialis than those of the superficial trapezius and biceps brachii (Fig. 5). Although the intra- and inter-operator reliabilities of the SWV measurements for the trapezius and biceps brachii were almost perfect, they were only substantial for the deeper supraspinatus and brachialis muscles (Table 3).

**Table 3:** Intra- and inter-operator reliability of shear wave velocity (SWV) calculated by measuring skeletal muscle on color Doppler shear wave imaging

Muscle	SWV (m/s)	ICC (1.1)	ICC (2.1)
Trapezius	i: 3.65 ± 0.06	0.864	0.923
	ii: 3.66 ± 0.08	0.924	
Biceps brachii	i: 3.76 ± 0.08	0.87	0.885
	ii: 3.80 ± 0.07	0.913	
Supraspinatus	i: 3.68 ± 0.20	0.796	0.728
	ii: 3.70 ± 0.21	0.728	
Brachiallis	i: 3.78 ± 0.12	0.789	0.777
	ii: 3.70 ± 0.21	0.742	

i: observer 1, ii: observer 2

CV tended to be greater in the deep SSP and brachialis than in the superficial trapezius and biceps brachii.

## Discussion

Because CD SWI is fundamentally different from SWE, we compared these two methods and assessed the precision and reliability of CD SWI. The results obtained using phantoms showed that CD SWI yields the same measurements and has similar high precision and reliability as SWE.

However, even CD SWI tended to produce varying values in deeper layers (2–4 cm). The highest CV was 6.5–6.8%, and the standard deviation of SWV was 0.01–0.03 m/s. The error was exceedingly small and was unlikely to cause a problem when taking measurements of the living body.

Baumer et al. previously measured the SWV in three phantoms of varying stiffness using the SWE function of the Acuson S3000 ultrasound system (Siemens, California, USA) and compared the measurements obtained by two different operators [17]. The ICC for intra-operator reliability (1.1) was 0.99, whereas that for inter-operator reliability (2.1) was 0.68. In the present study, the CD SWI showed an intra-operator reliability similar to that of the Acuson S3000 measurements, although the inter-operator reliability was higher. The CV of 1.55 ± 0.004 m/s SWV measured in the softest phantom was 0.25% and that of the 3.97 ± 0.02 m/s SWV in the stiffest phantom was 0.5%. Although the CV was greater with CD SWI, a similar tendency was observed: when the stiffness of the phantom increased, the variation in the measured values and CV increased.

Generally, the amplitude of the shear waves increases in stiff materials. This increased variation in the measurements of stiffer phantoms may be caused by increased noise, reflections, and other waves inside the phantoms. Dillman et al. previously evaluated the SWV in two phantoms (soft and stiff) at three different depths (1.0, 2.5, and 4.0 cm) using two SWE systems [18]: Acuson S3000 (Siemens, California, USA) and Aixplorer (Supersonic Imaging, Aix-en-Provence, France). They found that the SWV at the superficial layers was significantly (*P* < 0.0001) higher than that at the deep layers when measured with Acuson S3000, but was significantly (*P* < 0.0001) lower when measured with Aixplorer.

The most obvious cause of increased SWV in superficial layers is precompression from the pressure of the probe, as reported in studies concerning elastography in the mammary region [19].

The dead weight of phantoms is thought to be a cause of increased SWV in the deeper layers. This effect of weight likely increases with depth, subsequently producing a higher SWV.

In CD SWI, which was used for evaluation in the present study, shear waves in the phantoms were displayed visually, and the ability to select the appropriate location may have led to equal or greater precision and reliability than those previously reported with SWE. The reason that precision was retained with CD SWI compared to that with SWE at depths where shear waves are easily damped may be the ability to adjust the frequency applied to the tissue.

Previous studies have shown that CD SWI provides highly reliable measurements of living muscles. In measurements of the trapezius muscle, ICCs for intra- (1.1) and inter-operator reliability (2.1) have been reported to be 0.91 and 0.83, respectively [10–12]. In the present study, CD SWI exhibited good intra- and inter-operator reliability when measuring the trapezius, biceps brachii, supraspinatus, and brachialis muscles (Table 4). Furthermore, better reliability was obtained in deeper layers in the present study than in previous reports; this may be a unique advantage of CD SWI that deserves special mention.

**Table 4:** Previous reports of intra- and inter-operator reliability of shear wave velocity calculated using color Doppler shear wave imaging (CD SWI) and shear wave elastography

Muscle	ICC		References
	CD SWI	Past reports	
Trapezius	0.864–0.924	0.994	Ishikawa et al. [24]
		0.87–0.97	Leong et al. [25]
Biceps brachii	0.870–0.913	0.91	Alfuraih et al. [17]
		0.935–0.957	Chen et al. [18]
Supraspinatus	0.796–0.728	0.70–0.80	Rosskopf et al. [20]
		0.931–0.998	Muraki et al. [28]
Brachiallis	0.789–0.742	No report	

Alfuraih et al. [20] previously assessed the biceps brachii. In this study, the participants were asked to hold their elbow at a 90° angle, and a LOGIQ E7 (GE Healthcare, Milwaukee, USA) SWE system was used to scan the biceps brachii. High reliability was obtained with a reported intra-operator ICC of 0.91 (0.82–0.96). Chen et al. [21] also reported high reliability, with intra-operator reliability ICCs of 0.869–0.957 and an inter-operator reliability ICC of 0.935, using the Acuson S3000 SWE system to take measurements with the elbow fully extended or bent at 30°. In both studies, measurements were obtained using a probe held longitudinally to the biceps brachii. It has further been reported that this approach provides better reliability than the transverse orientation [22]. In the present study, when CD SWI measurements were made using a longitudinal orientation with the subject’s elbow fully extended, the reliability was as high as that reported in previous studies, and a longitudinal orientation was appropriate for measuring skeletal muscle elasticity.

In a study by Rosskopf et al., the Acuson S3000 (Siemens, California, USA) was used to obtain measurements in the supraspinatus, with the probe held at 90° to the muscle [23]. The reliability was good, with intra-operator reliability ICCs of 0.700–0.800, and an inter-operator reliability ICC of 0.894.

Our method obtained better results because when the muscle is observed in the longitudinal direction while using the B-mode image, the shear wave propagates in a state of less strain, as the skeletal muscle is aligned in the longitudinal direction; therefore, this direction is considered optimal for measuring elasticity. This method has been reported in the past [14] and is considered to be an advantage of the CD SWI.

Because the present study revealed that the CD SWI measures the trapezius, biceps brachii, and trapezius with a higher reliability than that reported previously, we attempted to compare the results for the brachialis (in which similarly high precision and reliability were obtained) with the results of previous reports. The brachialis flexes the elbow and helps stabilize the upper arm [15]. As it is attached to the elbow joint capsule, we can assume that it is of greater interest to physicians in diagnosing conditions related to the locomotory organs to understand the clinical implications of its stiffness. However, there have been almost no reports of the reliability of SWV measurements of the brachialis. SWE tends to give variable measurements [16] because the brachialis lies under the biceps brachii and over the humerus, which is believed to explain the lack of relevant reports. As high reliability was obtained with CD SWI, with ICCs ranging from 0.742 to 0.789, satisfactory evaluation of the brachialis may be possible. The ability to perform assessments under the conditions described above (i.e., in deep muscles overlying a strongly reflecting bone), under which precise and reliable measurements with conventional SWE would be difficult, is thought to be one of the advantages of CD SWI over conventional SWE.

Past reports have further measured the stiffness of the supraspinatus [24, 25] to assess rotator cuff tears and inflammation [26]. In one report, the biceps brachii was associated with Parkinson’s disease stage [27], indicating that the usefulness of elastography is expected to increase in the future.

Overall, the reliability of CD SWI to measure the muscles mentioned above is not inferior to that of conventional SWE, and it seems to show significant promise for clinical applications in the future. However, as its reliability was slightly lower in the deep supraspinatus and brachialis muscles compared with that in the superficial trapezius and biceps brachii muscles, and as CV increased with depth in the phantom tests, its limitations must be considered when applying it in a clinical setting. Another reason for this is the damping of the shear waves.

The present study had several limitations which should be mentioned. Firstly, participants were young and exclusively male, and the low number of samples made statistical investigation difficult. Further, the muscles were at rest during measurements, and we only investigated the superficial muscles in the upper body.

Nevertheless, in the present study, we were able to clarify the precision and reliability of the CD SWI in measuring SWV. The CD SWI is still in development;further studies are warranted.

We believe that this technique could be suitable for evaluating the effectiveness of treatment in patients with shoulder stiffness, muscle spasticity after stroke, and muscle disease.

## Conclusion

CD SWI, a new type of elastography that allows visualization of shear waves, is a valid method to measure SWV. CD SWI showed high precision and reliability of measurement as SWE in both phantoms and live subjects, although its precision and reliability were not as good in deeper layers as in superficial layers.

## Data Availability

All data generated or analysed during this study are included in this published article.
